# Rapid Palatal Expansion to Treat Nocturnal Enuretic Children: a Systematic Review and Meta-Analysis

**Published:** 2015-09

**Authors:** Karim Poorsattar-Bejeh Mir, Arash Poorsattar-Bejeh Mir, Morvarid Poorsattar-Bejeh Mir, Maziar Moradi-Lakeh, Pouya Balmeh, Kamran Nosrati

**Affiliations:** 1 Dept. of Pediatrics, Amir Mazandarani General Hospital, Mazandaran, Iran.; 2 Dental Materials Research Center, School of Dentistry, Babol University of Medical Sciences, Babol, Iran.; 3 Dept. of Orthodontics, University of Alabama, Birmingham, USA.; 4 Dept. of Community Medicine, Iran University of Medical Sciences, Tehran, Iran.; 5 Undergraduate Student, School of Dentistry, Babol University of Medical Sciences, Babol, Iran.; 6 Dept. of Maxillofacial Surgery, School of Dentistry, Babol University of Medical Sciences, Babol, Iran.

**Keywords:** Cross bite, Enuresis, Meta-analysis, Rapid palatal expansion, Systematic review

## Abstract

**Statement of the Problem:**

Refractory nocturnal enuresis possesses a heavy psychosocial burden for the affected child. Only a 15% spontaneous annual cure rate is reported.

**Purpose:**

This patient-level meta-analysis aimed to evaluate the efficacy of rapid palatal expansion to treat nocturnal enuresis among children.

**Materials and Method:**

A sensitive search of electronic databases of PubMed (since 1966), SCOPUS (containing EMBASE, since 1980), Cochrane Central Register of Controlled Trials, CINAHL and EBSCO till Jan 2014 was performed. A set of regular terms was used for searching in data banks except for PubMed, for which medical subject headings (MeSH) keywords were used. Children aged at least six years old at the time of recruitment of either gender who underwent rapid palatal expansion and had attempted any type of pharmacotherapy prior to orthodontic intervention were included.

**Results:**

Six non-randomized clinical trials were found relevant, of which five studies had no control group. Eighty children were investigated with the mean age of 118 (28.12) months ranged from 74 to 185 months. The median time to become completely dry was 2.87 months [confidence interval (CI) 95% 2.07-2.93 months]. After one year, the average rate of becoming complete dry was 31%. The presence of posterior cross bite [relative risk (RR): 0.31, CI 95%: 0.12-0.79] and signs of upper respiratory obstruction during sleep (RR: 5.1, CI 95%: 1.44-18.04) significantly decreased and increased the chance of improvement, respectively. Meanwhile, the other predictors did not significantly predict the outcome after simultaneous adjustment in Cox regression model.

**Conclusion:**

Rapid palatal expansion may be considered when other treatment modalities have failed. The 31% rate of cure is promising when compared to the spontaneous cure rate. Though, high-level evidence from the rigorous randomized controlled trials is scarce (Level of evidence: C).

## Introduction

Nocturnal enuresis which is defined as involuntary loss of urine after the age of five is not an uncommon problem. About 15% of children are affected at this age. This rate would dramatically decrease to 5% and 1-2% at the ages of 10 and 15 years.[1-2] A report from Iran indicated that 17.5% of first-grade schoolers were affected and similar to the other investigations, it is exceeding more frequent in male patients than the female patients.[3-5]

Monosymtomatic nocturnal enuresis, the absence of or subtle daytime symptoms, constitutes 80% of cases with nocturnal enuresis. These cases can be classified into either primary (i.e., never achieved long-lasting dryness) or secondary (i.e., dryness has been achieved for at least six months before enuresis begins).[6] An annual spontaneous rate of 15% is estimated without any medicopharmacologic intervention.[7] One might note that nocturnal enuresis should not be confused with nocturia, which is the frequent night awakening to void.[8]

The aetiology of monosymtomatic enuresis is not clearly understood.[9]Neveus hypothesized that it is mainly a sleep disorder and low arousability, nocturnal polyuria and detrusor hyperactivity are the other contributors to the nocturnal enuresis. Neveus attributed the high arousal threshold of enuretic patients to disturbance of the upper pons.[9]Furthermore, sleep disorders, sleep-disordered breath (SDB), and psychological abnormalities may be the major accompaniers.[10-12] Up to 80% of enuretic children have concurrent sleep apnea.[13]Sleep patterns of enuretic and non-enuretic children are the same.[11]Nevertheless, the major problem is associated with the deep sleep and low arousability or high arousal threshold among enuretic children.[9-12] Nocturnal enuresis could occur in any stage of sleep. Antidiuretic therapy effectively can reduce the wet nights, however, sleep pattern may remain unchanged.[14]

It is shown that the severity of nocturnal enuresis and obstructive sleep apnea are correlated.[13] As previously shown, adenotonsillectomy, by reduction of nocturnal resistance airflow, may alleviate enuresis in children with SBD and hypertrophic tonsils.[15]

Generally, the proposed treatment modalities are motivational therapy, bladder training, fluid management, night alarms and pharmacological agents such as desmopression and tricyclic antidepressants. Based on most meta-analyses and clinical trials, however, the plateau of evidences is just in favor of night alarms and pharmacotherapy with either oral desmopression or imipramine.[6]

Nonetheless, a few percent of enuretic children may remain unresponsive that brings a heavy psychosocial burden for both the child and family. In addition to the standard and accepted modalities, alternative methods such as mandibular advancement and complementary alternative medicine such as eletro-acupunture are suggested for refractory enuresis.[16-17]Moreover, there are some promising outcomes regarding the management of such resistant cases implementing an orthodontic device by rapid increasing of maxillary width within 10-14 days (i.e., average five millimeters), so-called rapid palatal expansion (RPE).[18-21] This technique dates back to 19^th^ century (1860), when Angel successfully treated posterior crossbite which became more popular with altering popularity and declined in various eras.[22-23] Maxillary bone articulates with 10 other craniofacial bones, hence, maxillary expansion would influence the structures of temporo-mandibular joints (TMJ) and those adjacent nasal and pharyngeal spaces. In addition to the crowding and cross bites, RPE can be applied to improve nasal flow resistance, conductive hearing loss, TMJ dysfunctions and asymmetric position of the condyle.[22, 24-26] Notably, increasing the nasal chamber radius by expanding the nasal floor will increase the nasal volume by a power of four (Poiseuille’s Law).[25]

There is no meta-analysis of existing literature regarding the effect of RPE on children nocturnal enuresis. We opt to perform a meta-analysis of pooled data from previous researches to discover the real efficacy of this unique treatment modality with the increased power of analyses. In addition, the effect of various unmet potential predictors such as age, gender or dento-skeletal morphology may be better explored. 

## Materials and Method

*Search strategy, Inclusion criteria and Study identification*

A sensitive search of electronic databases of PubMed (since 1966), Scopus (containing Embase, since 1980), Cochrane Central Register of Controlled Trials, CINAHL and EBSCO till February 2012 was performed which was updated till Jan 2014. The Google Scholar was also searched for probably non-indexed available articles in other sources. Also, gray literature was sought by hand search and Gateway Medline was evaluated for meetings and conference abstracts. A set of regular terms was used for searching in data banks except for PubMed, for which medical subject headings (MeSH) keywords were used. 

Search strategy using MeSH keywords were “rapid [All Fields] and ("palatal expansion technique "[MeSH Terms] or ("palatal"[All Fields] and "expansion "[All Fields] and "technique"[All Fields]) or "palatal expansion technique"[All Fields] or ("maxillary" [All Fields] and "expansion"[All Fields]) or "maxillary expansion "[All Fields]) and ("enuresis" [MeSH Terms] or "enuresis "[All Fields])”. We were interested in original articles; case reports and case series which were published in English language. Also, the searching was enhanced by bibliographic survey of the existing review articles.

*Data Extraction and Identification of Eligible Studies*

Four authors (APB, KBP MPB and PB) independently performed the literature review and decision was made after final agreement with consultation with the other author (MML) in the case of disagreement between them. Inclusion criteria were to enrol the published investigations of rapid palatal expansion to treat nocturnal enuresis as the main outcome. To be included, participants should be at least six years old at the time of recruitment of either gender , should at least be followed up for six months after commencing the treatment and should have had attempted any type of pharmacotherapy previously. To assess the quality of non-randomized clinical trial a modified Newcastle Ottawa Scale (NOS) was applied to qualify the final non-randomized enrolled articles.[27]

*Subgroup Analysis*

Demographic data were obtained. Also, information about frequency of bed wetting, signs and symptoms of upper airway obstruction (e.g., snoring, open mouth during sleep and sleep apnea), parental divorce (i.e., indicator of pre-existing stressor), skeletal Angle’s classification (i.e., Angle class I-III dento-skeletal relationship), presence of crossbite (i.e., jaws width discrepancy which leads to circumferential malpositioning of the first permanent molar with regards to its antagonist), average palatal expansion, study methodology, response rates (i.e., responders, partial responders and non-responders), time to become completely dry or improved, follow ups and enuresis type (i.e., primary or secondary) were gathered. Response rate was considered with two binominal modes; complete or partial response versus no response and complete response versus partial or no response. Correlations of dento-skeletal occlusion with triple response categories were first evaluated by means of Gamma ordinal statistics, thereafter two-by-two cross-tabulation was performed between the binominal Angle classes (I vs. II, III or II vs. I, III or III vs. I, II) and two before-mentioned binominal response categories. These comparisons were reported by either a Pearson Chi-square or Fisher’s exact test preceded by a Cochrane’s test for the adjustment of nominal confounder. Age variable was first entered as a continuous numeric data. Afterwards, average age obtained from the receive operating characteristic (ROC) curve and a dichotomous age predictor was entered to the model. Also a cut-point of ten was evaluated as previously reported by Schutz-Fransson.[21] We defined severe nocturnal enuresis when the child wet the bed more than four nights a week. Comparisons of wet nights between triple-response categories were evaluated with the analysis of variance (ANOVA) which was adjusted with a Browns-Forsythe statistics. Further multiple comparisons were accomplished with a Games-Howell adjustment. Overly, an average was entered when data were displayed with a narrow range of wet nights.

*Statistical Methods*

The nominal data were expressed with frequencies (%) and the numeric data were shown as mean (standard deviation). Meta-analysis was accomplished applying an individual patient data (IPD) method. Cumulative portions of survival at 12^th^ month after the beginning of the study were calculated and plotted by the Kaplan-Meier analysis. There was not a common and certain definition for partial response between the studies; hence, complete responders were mainly analyzed against partial and non-responders. A Cox-regression model was also built with a forward conditional method to investigate the effect of predictors on treatment outcome and odds ratios were displayed with a two-sided 95% confidence interval (CI). In addition, a further subgroup analysis was carried out to determine whether the effect of variables was influenced by the interaction of the others or not. The mean wet nights/ week was compared before and after RME considering stable responses around six to twelve months after the initiation of orthodontic treatment with a Wilcoxon signed rank test. Publication bias and heterogeneity of final included studies were checked by Begg and Manzumdar adjusted rank correlation test and I[2] statistics, respectively. Further data were displayed by Funnel plot and Forest plot.

## Results

*Systematic Literature Search*

**Figure 1:**
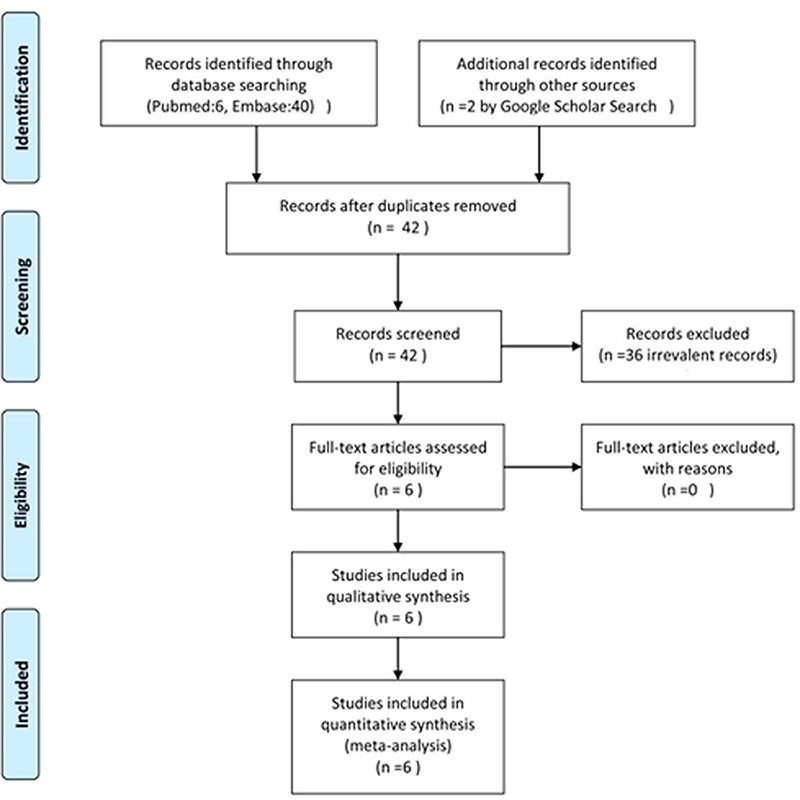
PRISMA flow diagram of the searched studies.

Due to a few existing studies; we extended our inclusion criteria to all types of published and unpublished studies. Moreover, follow up duration of at least 6 months were excluded from the primary exclusion criteria. Six clinical trials were found relevant (Figure 1, Table 1).

**Table 1:** Details of Final Enrolled Studies in the Meta-Analysis

**Study** **(Study year, ** **Reference no.)**	Patients No., Mean age (SD) *	**Study Type**	**Response** **(Time since the ** **onset of therapy)†**	**Gender** **(Male: ** **Female)**	**Enuresis** **(Type)** **††**	**Frequency ‡** **Before- After**	**Expansion ** **Rate (mm), ** **Mean**	**Follow- Ups** **(Months)**	**Quality** **‡‡**
Timms (1990,17)	10, 128.6(38.16)	Non-randomized, Non-controlled Prospective Clinical Trial	CR:10 (1^st^ to 4^th^ month)	7:3	8:P, 2:S	2.5 (1.75)- 0.0(0.0) (1^st^ to 4^th^ month)	6-10, **N/A	**N/A	3
Kurol *et al.* (1998,18)	10, 128.6(20.62)	Non-randomized, Non-controlled Prospective Clinical Trial	CR:4, PR:3, NR:3 (1^st^ month)	8:2	9:P, 1:S	6.3 (1.49)-2.8(2.76) (1^st^ month)	3-5,4.1	12	3
Akhavan-Niaki & Farbod (2000,28)	10, 91.90(10.44)	Non-Randomized, Non-controlled Prospective Clinical Trial	CR:1,PR:3, NR: 6 (1^st ^month) CR:6, PR:4 (3^rd^ month)	6:4	P	3.05 (1.28)- 2.0(1.33) (1^st^ month) 0.7-1.06 (3^rd^ month)	4-7,5.83	3	3
Usumez *et al.* (2003,19)	8, 112.50 (10.99)	Non-randomized, Non-controlled Prospective Clinical Trial	PR:7, NR: 1 (1^st^ month)	6:2	P	7(0)- 2.17 (1.47) (1^st^ month) 2.15(1.68) (6^th^ month) 2.87(1.75) (8^th^ month)	3-6,3.9	8	3
Al Taai *et al.* (2005,25)	19, **N/A	Non-Randomized, Controlled Prospective Clinical Trial	CR:13, PR:6 (20^th^ day)	**N/A	P	7.0(0.0)-0.42 (0.69) (20^th^ day)	**N/A	9-10	3
Schutz-Fransson & Kurol (2008,20)	23, 121.35 (29.48)	Non-randomized , Non-controlled Prospective Clinical Trial	CR:6, PR: 5, NR:12 (1^st^ month)	18:5	P	6.2(1.83)-N/A	5-8,6.5	12,120	3

* Ages are reported in month, †CR: Complete Responder, PR: Partial Responder, NR: Non-Responder, ††P: Primary, S:Secondary, ‡ Means (Standard deviation) for frequency of wet nights/week before and after maxillary‡‡Assessed with Ottawa New-Castle Quality Scale (obtained stars out of 9 maximum possible stars), **N/A: not available

**Figure 2:**
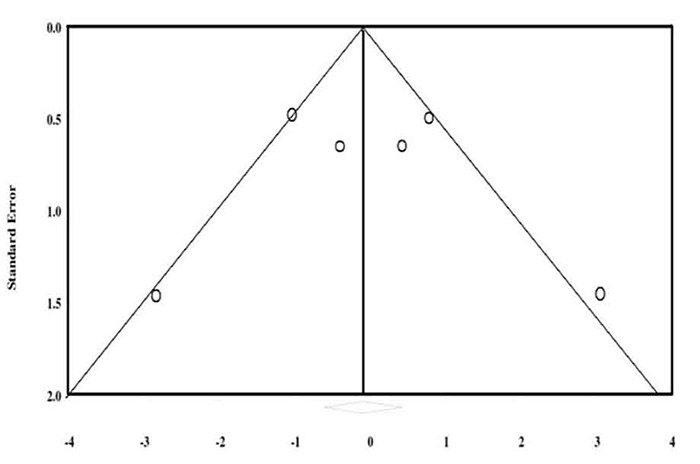
Funnel plot to display the publication bias

All included studies were case series. Final recruited studies were conducted in England, Sweden, Iran, Turkey and Iraq between 1990 and 2008. Emails were sent to three authors for additional data; one responded yet could not provide the requested data. No publication bias was found (tau=0.13, *p*=0.7) (Figure 2).

**Figure 3:**
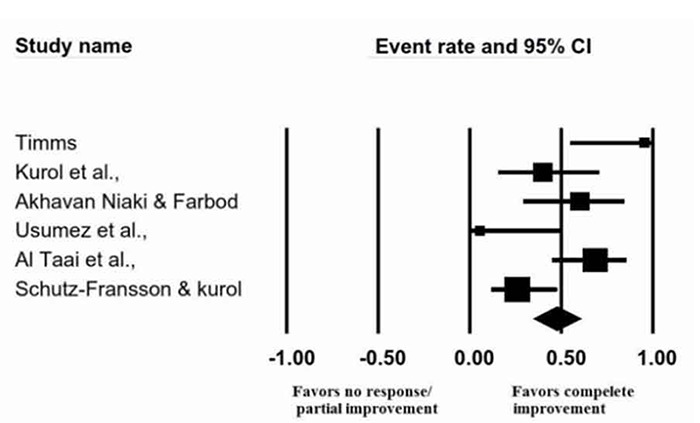
Meta-analysis of pooled data to display the effectiveness of RPE to cure NE (complete dryness).

The effect size calculated by random model was 0.49 (CI 95% 0.26-0.73) (Figure 3). Statistically heterogeneity was observed between included studies (I[2]=%69, *p*= 0.006). Relative weights for studies by Timms, Kurol *et al.*, Akhavan Niaki and Farbod, Usumez *et al.*, Taai *et al.*, and Schutz-Fransson and Kurol were respectively 3.34%, 16.80%, 16.80%, 3.3%, 28.73% and 31.04%.

*Samples and Intervention’ Characteristics*

**Figure 4:**
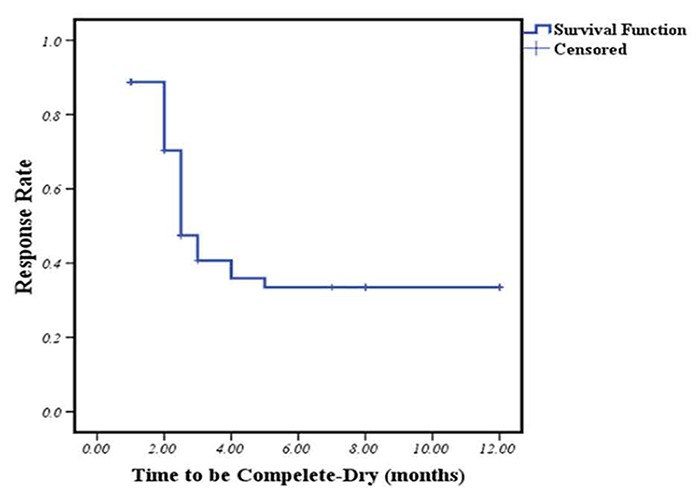
Kaplan-Meier survival curve to estimate the percent of dry children at 12th month after the commencing of orthodontic palatal expansion

Eighty children were entered in our study investigated with the mean age of 118 (±28.12) months ranged from 74 to 185 months. Descriptive and demographic data are presented in Table 2. All studies used a hyrax screw which was inserted in an acrylic expanding device. A rapid heavy force was delivered to the midpalatal suture by the activation of screw; hence, the suture was distracted and two palatal shelves were pushed to the sides and a diastema appeared between central incisors.[25] Device was kept in place after finishing the active phase (10-14 days) for a few months as a retainer for the retention phase to prevent further collapse. Thirty nine children (48%) responded to the treatment with the complete improvement, 25 children (32%) were partially improved and 16 children (20%) did not respond to the treatment. Median time to become completely dry was 2.87 months (CI 95% 2.07-2.93 months). The average rate of becoming completely dry a year after commencing the 10- to 14-day orthodontic expansion of maxilla was 31% (Figure 4).

A significant improvement was observed after six to 12 months of orthodontic maxillary expansion in terms of number of wet nights per week [5.63(2.14) to 1.58(1.82), *p*< 0.001]. Detailed data of the studies which were included in our meta-analysis and other predictors including age, gender, occlusion classification, upper airway obstruction, frequency of bed wetting , and parental divorce are shown in Table 1, 2.

**Table 2:** Descriptive and analytic data of recruited children and the significances to predict complete dryness at the end of trial.

**Variable**	Mean (±SD)^*^, **Frequency (%)**	**Comparisons’ Significance**	**Description**
Gender	Male 45 (56%)	X[2](1)=0.63, *p*= 0.43	The gender of 19 children (24%) was not identified in the context. No interaction was found between the age and gender of the participants to predict the improvement (*p*= 0.22)
	Female 16 (20%)		
Age (months)	118 (±28.12)	<120 vs. >120mo: X[2] (1)=0.04, *p*= 0.84	Cut point of 120 months was proposed by Schutz-Fransson and Kurol [21]
		<105 vs. >105mo: X[2] (1)=5.03, OR: 3.37, CI 95%: 1.14-9.93, *p*= 0.03	Cut point of 105 months was selected from the ROC curve
Occlusion^‡^	I 25 (31%)	I vs. II,III^†^NS**	Data for 19 children (24%) were not available
	II 33 (41%)	II vs. I,III^† ^NS**	
	III 3 (4%)	III vs. I,II^†^NS**	
UAO^‡‡^	Yes 56 (70%)	B: 1.62, OR:5.1, CI 95%: 1.44-18.04, *p*= 0.01	The presence of signs of upper respiratory obstruction during sleep increased the chance of resolution of NE by 5.1-fold
	No 16 (30%)		
C_x_^#^	Yes 14 (17%)	B: -1.18, RR: 0.31, CI 95%: 0.12-0.79, *p*=0.01 Adjusted for gender: Cochrane’s X[2](1)=5.09, *p*= 0.02	Data for 29 children (36%) were not available. It was more frequent in male children (Fisher exact test *p*= 0.004). Presence of posterior cross bite decreased the chance of complete resolution with 0.31-fold.
	No 37 (47%)		
Frequency^##^ (nights/week)	NR 6.33(±1.80)	<4 vs. >4 nights/wk.: X[2], OR=4.08 CI 95%: 1.24-13.43 *p*= 0.02 Adjusted for age (<105 vs. >105mo): Cochrane *p*= 0.04	Data from five studies, except for the Usumez’ study, [20] were taken into account. Wet nights of NR group was significantly higher than PR group (*p*= 0.02) and CR group (*p*< 0.001)
	PR 4.11(±2.62)		
	CR 3.72(±2.41)		
Parental Divorce	Yes 13 (16%)	*p*=1	41 children (51%) had clear report of this background
	No 28 (35%)		

†There were no significant correlations between either types of Angle classes (I vs. II, III; II vs. I, III; III vs. I, II) and response type (i.e., complete or partial response vs. no response; complete response vs. partial or no response, all P values were >0.2), except for a lower complete improvement rate observed in children with class III malocclusion with a marginal statistically significance (Fisher’s exact test *p*= 0.07).* standard deviation,** not significant ‡Angle molar relation, ‡‡upper airway obstruction, # cross-bite, ## CR: Complete Responder, PR: Partial Responder, NR: Non-Responder

## Discussion

A patient-level meta-analysis of rapid palatal expansion to treat refractory nocturnal enuretic children is presented. In commitment to a very recent review article by Holty and Guilleminault,[15] a significant improvement of wet nights per week may be expected by orthopaedic maxillary expansion. Holty and Guilleminault concluded that 51 enrolled enuretic children in the studies of Timms, Kurol *et al.*, Usumez *et al.*, and Schutz-Fransson and Kurol reported the remarkable reduced wet nights [5.6(2.4)-1.8(2.3), *p*< 0.001].[15]

*Possible explanation of findings*

As previously mentioned, a male gender tendency exists in the literature for the possible affection by nocturnal enuresis. Nevertheless, no contrasting pattern of improvement is observed between the male and female enuretic children. Similarly, no distinguishable model is reported from the previous researchers.[18-20,26] Our findings may be biased by a considerable portion of unreported information from two studies.[21, 26]

A lower spontaneous curing rate is expected when the disease is protracted.[2]Supported by our findings, the younger the child, the higher the chance to become dry. In commitment to Schutz-Fransson and Kurol who reported that a higher portion of children with complete improvement was younger than 10, we found a comparable cut-off point of 105 months.[21] As expected, milder cases, in terms of frequency of bed wetting, responded better than the more severe cases (Odds Ratio=4.08).

There are controversies considering the improvement of nasal airflow by RPE. Supported by Timms, Kurol and Al-Taaia clear benefit was expected in terms of reduced nasal resistance and increased nasal airflow,[18-19,26] Nevertheless, Schütz-Fransson and Kurol argued that nocturnal enuresis improvement was irrelevant of such modifications.[21] Rapid palatal expansion increases the maxillary width in a non-parallel wedge-shaped manner, with most increment in the anterior part. This is best explained by anatomic inhibitory effect of sphenoid bones located posteriorly to the palatine bones.[21] However, there is no consensus on which direction it acts most.[29-30] The presence of crossbite was found to have a confounding effect on improvement. It was expected since most attempts were exerted on normalizing the existing deficit instead of over treating the maxilla. In addition, children with posterior cross bite received the least benefit from RPE due to the substantial increase by non-equal widening, which was considerably higher in the anterior part of palate.[22]

A considerable portion of enuretic children was found to have concurrent sleep problems which was in agreement with a previous work by Barone *et al.*[13] With regards to Weirder reports, elimination of airway obstruction at nasopharyngeal or oropharyngeal level with either tonsillectomy or adenoidectomy or both may improve the nocturnal enuresis.[31-22] This finding was proved later by Cinhar and Weissbach.[33-34] Guilleminault *et al.* performed a cross-over clinical trial to compare adeno-tonsillectomy with maxillary/ bi-maxillary expansion to treat children with obstructive sleep apnea syndrome (OSAS) and concluded that there might be an additional gain with combined therapies. It should be emphasized again that the anatomical location of obstruction is critical to decide monotherapy or to perform such bitherapies.[35] Narrow hard palate and enlarged tonsils are present in majority of children with OSAS, hence bitherpay is rationale. Nevertheless, a child with isolated enlarge tonsils benefits mostly from adeno-tonsillectomy and isolated nasal obstruction demands an orthodontic treatment rather than a surgical approach. This fact may rationalize the heterogeneity of outcomes from various studies. In the present meta-analysis, the number of cases with exact report of such information was too low and further subgroups analyses were not applicable which were unreliable and low-powered. Of included children in current meta-analysis, a considerable portion exhibited improved breath pattern, nasopharyngeal airway dimensions and airflow.[18, 20, 26, 28] Reported by Timms, many children claimed easier nasal breathing after RPE.[18]Six out of ten children in Akhavan Niaki and Farbod investigation changed oral breath to nasal breath.[28] Establishment of a correlation between cessation of nocturnal enuresis and degree and pattern of nasal airflow is still a matter of debate.[19, 21] A plenty is still to be known about the effect of concomitant obstructive sleep apnea and improvement of nocturnal enuresis by RPE. Actually, inconsistent data regarding reporting the presence of concomitant obstructive sleep apnea and documented improvement of airflow assessed with a same method make it difficult to pool the existing data.

Enuresis has a high negative psychosocial burden. The feeling of helplessness of enuretic children highlights the magnitude and complexity of the problem.[36] Prolonged nocturnal enuresis adversely affects the coping, social competence, and school performance of enuretic children when compared to their normal peers.[37] Of interest, a negative correlation exits between the self-esteem of an enuretic child and the chance of treatment failure.[38] One should note that the considerable annual spontaneous resolution rate should not be approached in a dogmatic manner. When failure is deemed in future, a child could be earlier intervened and supported, because increased consequent stresses might prevent from the optimal outcomes even in the non-complex cases. Parental divorce as a psychological confounder was not contributory to the outcome. Previously, Desta *et al.*[12] reported a higher prevalence of parents’ separation among enuretic children. Meanwhile, they were not quite sure about the direction of correlations between the psychopathologies and enuresis.[12] Noteworthy, in contrast to the long lasting belief on the detrimental effect of psychological abnormalities; currently such abnormalities are approached as a result, rather than the cause of nocturnal enuresis.[39]

Fields *et al.*[40] found that the long- and normal-face individuals had similar tidal volume; yet nasal and oral passage did not share the same portion especially in long-face patients, who are mainly oral breathers.[40] Hence, it is probable that the long face patients suffer less from an obstruction located in the nasal cavity and may benefit mostly from the adeno-tonsillectomy. Different Angle classifications (i.e., I-III) were not different in terms of response rate in our study. Unfortunately, facial morphology was not provided by any of the included studies. Indeed, feature studies with higher sample size and complete list of confounders would better reveal the casual relationship. Moss, in his “Functional Matrix” theory stipulated that soft tissue would guide the growth direction of underlying hard tissue.[41] Hence, the anatomical level of obstruction in either the nasopharynx or oropharynx could probably influence the growth of maxilla and mandible. Recently, Nunes and Di Francesco reported a higher prevalence of class III malocclusion in isolated tonsillar enlargement and class II malocclusion in combined or isolated adenoid enlargement.[41] Uneven distribution of various classifications and under-reported or nondifferentiated sites of pharyngeal obstruction may contribute to somewhat different, albeit not significant correlations between the occlusion classifications and response type in our study which needs further investigations.

*Possible Mechanisms of Action and Safety Issues*

Historically, the first report of improvement of nocturnal enuresis with RPE belongs to Freeman in 1970, who investigated the effect of RPE on basal metabolism of mentally retarded children. He reported an unexpected side effect of abrupt cessation of nocturnal enuresis.[20] Surprisingly, a few years earlier Kunvari in 1964 had suggested that the correction of maxillary deformity would indirectly increase the lymph circulation of pituitary gland by means of bulging effect of maxillary vault beneath the floor of sella turcica.[42]It was further approved by Al-Taai *et al.*, who indirectly traced antidiuretic hormone (ADH) by measuring the plasma osmolality. They stipulated that the decreased plasma osmolality at the end of treatment of enuretic children with rapid palatal expansion was assumed to be consequence of increased ADH level.[26] Other possible mechanisms are improved neuromuscular coordination and lesser deep phase of sleep related to a higher oxygen saturation level derived by smoother and higher nasal airflow.[23] More recently, Sans Capdevila *et al.* found that higher brain natriuretic peptide is secreted in cases with either obstructive sleep apnea or enuresis. This might be due to the higher venous return and more dilated atrium accompanied by the airway obstruction and increased intra-thoracic negative pressure associated with posing a deep breath against a narrowed or collapsed airway.[43]

Importantly, RPE could increase the airflow.[19-21] This could improve nasal breathing and blood oxygen saturation.[21] The possible explanation of RPE in improving nocturnal enuresis may be correlated to obstructive sleep apnea. Hence, the latter suggested mechanism may be the corner stone of treatment and should be focused more on upcoming investigations.

Interestingly, sleep-disordered breathing and nocturnal enuresis share many non-orthodontic (e.g., adeno-tonsillectomy) and orthodontic (e.g., rapid palatal expansion) treatment options.[26] Focusing on concomitant obstructive sleep apnea in an affected child may guide the clinician to decide how to prioritize and individualize the treatment options.

Safety of RPE has been recently investigated by Li *et al.*[44] They qualified the brain blood supply by means of a dynamic perfusion computed tomography. They attributed the increased cerebral blood flow and cerebral blood volume to indirect forces of RPE through the circum-maxillary sutures, superior ophthalmic fissure, carotid sulcus, and foramen lacerum with subsequent vasodilatation of inner vasculature.[44] The child should be least six years old. This cut off point is warranted since urinary incontinent is not considered pathologic in a younger child and RPE can damage the naso-maxillary complex and make nasal deformity in younger preschoolers and toddlers.[45]

*Strengths, Limitations and Future Direction*

The current meta-analysis is a patient-level one with the individual patient data (IPD) method. To our knowledge, it is the first meta-analysis to assess the effect of rapid palatal expansion on nocturnal enuretic children. Individual patient data method has the advantages of higher final power and the ability to test the new hypotheses for casual relationships between the different variables of studies.[46] Also, this method enables the investigator to modify the confounding effects of some variables on the outcome, especially when such confounders were not considered in the original articles.

Moreover, aggregation bias is lessened, by which groups’ results cannot be confidentially associated to a particular patient. Nevertheless, the main disadvantage of this method is inability to access all detailed data.[46]

As presented before, data from included studies showed substantial heterogeneity. The analyses executed by subgroup analysis. Of note, few included studies and multiple subgroup analyses may possess false positive and false negative comparative outcomes. In addition, all of included researches lacked simultaneous control group which also had not random allocation designs. This drawback is also evident in poor obtained quality scores. These could be addressed as main limitations of this meta-analysis. Information of some variables such as familial history of enuresis, gender, exact and average bed wetting per night and per week were not reported in all researches. This lowered the powered the comparisons. Hence care should be taken into account interpreting the data of the current meta-analysis especially for those with considerable missing data. 

As another limitation, our analyses suffered from a right-censoring due to heterogeneous durations of follow ups. Our suggestions for the future works may be addressed to more powered randomized controlled trials with adequate sample size and treatment arms, and also concurrent polysomnographic study with special attention to the brainstem and ascending reticular arousal system (ARAS) activity and 3-combined maxillary expansion and mandibular advancement. 

## Conclusion

In conclusion, rapid palatal expansion increases the chance of improvement by 2.06-fold when compared to 15% annual spontaneous improvement rate. The presence of posterior cross bite (relative risk: 0.31) and signs of upper respiratory obstruction during sleep (relative risk: 5.1) significantly decreased and increased the chance of improvement, respectively. The younger the children (<105 months), the higher the success rate after rapid palatal expansion (odd ratio: 3.37).

Rapid palatal expansion to treat nocturnal enuresis can only be suggested when pharmacologic medication and nigh alarm are being attempted with no remarkable outcome. Importantly, refractory enuretic children should be checked for concurrent presence of obesity and obstructive sleep apnea. [43] By this age, the child can play an active role through the treatment. Indeed, any treatment modality may be efficient when accompanied by child’s willingness. It is noteworthy to mention that the current level of evidences lack enough support to recommend such alternative method to treat nocturnal enuretic children; hence, it can be suggested when other interventions have failed (Level of evidence: C). 
